# Body roundness index is related to the low estimated glomerular filtration rate in Chinese population: A cross-sectional study

**DOI:** 10.3389/fendo.2023.1148662

**Published:** 2023-03-28

**Authors:** Yue Zhang, Wenxing Gao, Rui Ren, Yang Liu, Binqi Li, Anping Wang, Xulei Tang, Li Yan, Zuojie Luo, Guijun Qin, Lulu Chen, Qin Wan, Zhengnan Gao, Weiqing Wang, Guang Ning, Yiming Mu

**Affiliations:** ^1^ Department of Endocrinology, the First Clinical Medical Center of Chinese People’s Liberation Army General Hospital, Beijing, China; ^2^ Medical School of Chinese People’s Liberation Army, Beijing, China; ^3^ School of Medicine, Nankai University, Tianjin, China; ^4^ Department of Endocrinology, The First Hospital of Lanzhou University, Lanzhou, Gansu, China; ^5^ Zhongshan University Sun Yat-sen Memorial Hospital, Sun Yat-sen University, Guangzhou, Guangdong, China; ^6^ Department of Endocrinology, The First Affiliated Hospital of Guangxi Medical University, Nanning, Guangxi, China; ^7^ Department of Endocrinology, The First Affiliated Hospital of Zhengzhou University, Zhengzhou, Henan, China; ^8^ Union Hospital, Tongji Medical College, Wuhan, Hubei, China; ^9^ Department of Endocrinology, Affiliated Hospital of Luzhou Medical College, Luzhou, Sichuan, China; ^10^ Department of Endocrinology, Dalian Municipal Central Hospital, Dalian, Liaoning, China; ^11^ Ruijin Hospital, Shanghai Jiao Tong University School of Medicine, Shanghai, China

**Keywords:** chronic kidney disease, body roundness index, visceral obesity, cross-sectional study, estimated glomerular filtration rate

## Abstract

**Background:**

Kidney disease is related to visceral obesity. As a new indicator of obesity, body roundness index (BRI) has not been fully revealed with kidney disease. This study’s objective is to assess the relationship between estimated glomerular filtration rate (eGFR) and BRI among the Chinese population.

**Methods:**

This study enrolled 36,784 members over the age of 40, they were from 7 centers in China by using a random sampling method. BRI was computed using height and waist circumference, eGFR ≤ 90 mL/min/1.73 m^2^ was considered to indicate low eGFR. To lessen bias, propensity score matching was employed, multiple logistic regression models were utilized to examine the connection between low eGFR and BRI.

**Results:**

The age, diabetes and coronary heart disease rates, fasting blood glucose, and triglycerides were all greater in participants with low eGFR. The BRI quartile was still positively connected with low eGFR after controlling for confounding variables, according to multivariate logistic regression analysis. (OR [95%CI] Q2:1.052 [1.021-1.091], OR [95%CI] Q3:1.189 [1.062-1.284], OR [95%CI] Q4:1.283 [1.181-1.394], P trend < 0.001). Stratified research revealed that the elders, women, habitual smokers, and those with a history of diabetes or hypertension experienced the connection between BRI level and low eGFR. According to ROC, BRI was able to detect low eGFR more accurately.

**Conclusion:**

Low eGFR in the Chinese community is positively connected with BRI, which has the potential to be used as an effective indicator for screening kidney disease to identify high-risk groups and take appropriate measures to prevent subsequent complications.

## 1 Background

Global public health is challenged by chronic kidney disease (CKD). The projected global prevalence rate of CKD in 2017 was 9.1%. it has been reported to be closely related to the occurrence of multiple diseases, bringing great economic burden and public health pressure (1). Due to the insidious occurrence and poor prognosis of chronic kidney disease, early detection of renal function decline has been the focus of people’s attention. eGFR is currently used to identify renal insufficiency in clinical practice.

Over the past 30 years, the prevalence of obese individuals has significantly risen worldwide (2). Obesity, particularly visceral obesity, has been linked to the beginning and development of CKD (3). The most popular method for determining obesity is body mass index (BMI) (4), but it has drawbacks because it does not account for how a person’s fat is distributed. The new obesity measurement index BRI developed by Thomas (5) has higher predictive power for visceral adipose tissue (VAT) percentage than BMI, and has been demonstrated to be linked to metabolic syndrome (6), non-alcoholic fatty liver disease (7) and other diseases are closely related.

Few research, meanwhile, have examined the link between BRI and chronic renal disease. In order to assess the relationship between BRI and low eGFR, we gathered data from 38361 persons in China for this study. The goal of this study was to provide evidence for the early prevention and management of CKD and to identify high-risk patients as early as possible.

## 2 Methods

### 2.1 Participants and study design

We obtained data from a prospective cohort study of cancer risk assessment in Chinese diabetic patients and non-diabetic people based on the community population (8), it received approval from Shanghai Jiao Tong University School of Medicine’s Ruijin Hospital’s Clinical Research Ethics Committee (No. 2014-25). Prior to taking the survey, each respondent provided written informed consent, which was carried out in accordance with the Helsinki Declaration. From May to December of 2011, data were gathered.

**Figure 1:**
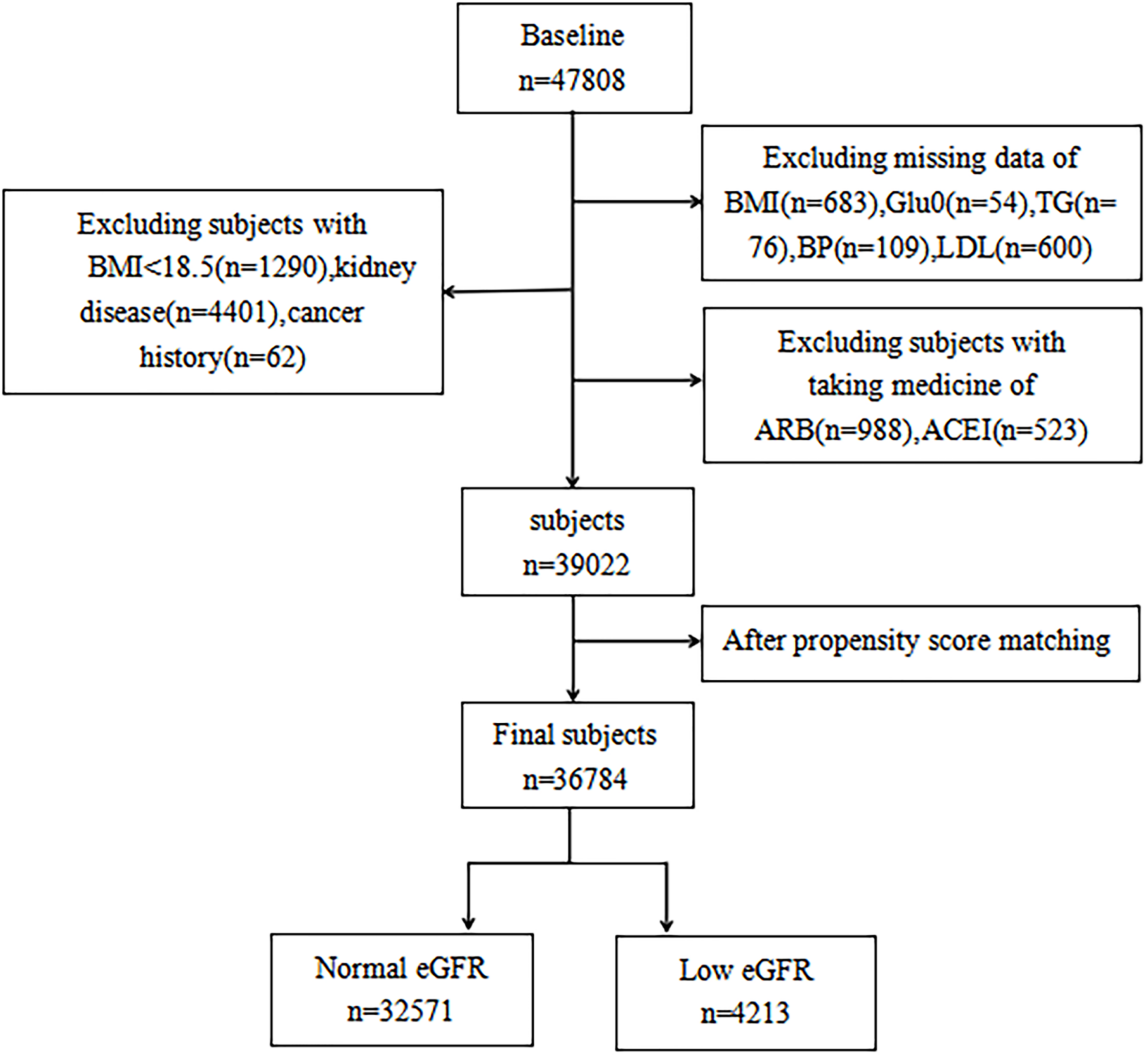
Flow chart of study population.

In seven geographically varied regional centers in China, a total of 47,808 participants over the age of 40 were registered using multi-stage stratified cluster random sampling (Dalian, Wuhan, Lanzhou, Zhengzhou, Luzhou, Shanghai, and Guangzhou). Propensity matching was employed to lessen potential bias, considering the variances in baseline traits between the two groups. Participants having a primary kidney disease diagnosis, a history of cancer, past antihypertensive drug use (angiotensin-converting enzyme inhibitors or angiotensin-receptor blockers), or insufficient data were excluded from the study. In the end, there were 36,784 participants (11,546 men and 25,238 women) (**Figure 1**).

### 2.2 Data collection and measurements

Trained investigators used standardized questionnaires to collect basic information about the participants, including demographic information (e.g., sex, age), lifestyle information (e.g., smoking, alcohol consumption), and disease history (e.g., kidney failure and tumors). Weight, height, waist measurement (WC), blood pressure, and other anthropometric measurements are included. Before collecting the measures, the patients were asked to remove their clothing and shoes. The height measurement is accurate to 0.1 cm and is done with a rangefinder. To accurately determine your weight to the nearest 0.1 kg, use a digital standing scale. Three readings of systolic blood pressure (SBP) and diastolic blood pressure (DBP) were taken with a mercury sphygmomanometer, and the average was taken. Prior to measurement, each subject was instructed to remain still for at least 5 minutes. While trained investigators measured WC, participants had to stand up straight, while dropping their arms naturally, and keeping their feet together. When exhaling, the exact halfway between the iliac crest’s topmost border and the bottom of the thorax is 0.1 cm (9).

A fast of 10 hours was followed by the collection of morning urine and fasting blood samples by qualified inspectors. Chemiluminescence immunoassay was used to measure the amounts of urinary albumin and creatinine, fasting blood glucose (FBG), glycated hemoglobin (HbA1c), triglycerides (TG), total cholesterol (TC), two hours postprandial blood glucose (PBG), high-density lipoprotein cholesterol (HDL-C), low-density lipoprotein cholesterol (LDL-C), alanine transferase (ALT), glutamyl transferase (γ-GGT), and aspartic acid transferase (AST).

### 2.3 Definition of variables

Weight divided by the square of height yields BMI (kg/m^2^). This formula is used to compute BRI: 364.2-365.5*(1-[WC(m)/2π]^2^/[0.5*height(m)]^2^)½ ^(5)^. Height and WC are both expressed in meters. Waist-to-Hip Ratio (WHR) and waist-to-Height Ratio (WHtR) are, respectively, WC divided by hip circumference and WC divided by height. No smoking, occasional smoking (less than one cigarette per day or seven cigarettes per week), and regular smoking were considered to be the three types of smoking (at least one cigarette per day). No drinking, occasional (less than once a week), and regular drinking were the three categories used to categorize drinking behaviors (at least once a week for more than six months).

Using the Collaborative Equation for the Epidemiology of Chronic Kidney Disease (CKD-EPI eGFR) (10) to estimate the eGFR. Participants were split into two groups: eGFR < 90 mL/min/1.73 m^2^ for low eGFR and eGFR ≥ 90 mL/min/1.73 m^2^ for normal eGFR. Blood pressure was divided into the normal group (SBP < 120 and DBP < 80), prehypertension group (120 ≤ SBP < 140 and/or 80 ≤ DBP < 90), and hypertension group (SBP ≥ 140 or DBP ≥ 90).

### 2.4 Statistical analysis

Because eligible participants in the two sets of eGFR had different baseline characteristics, propensity score matching was used to minimize bias. All covariates were matched at a ratio of 1:6, with caliper widths equal to 0.05 of the propensity score Logit’s standard deviation. Continuous variables were described by mean and standard deviation (SD), or median and (25th percentile, 75th percentile) depending on whether they were regularly distributed. T-tests or Mann-Whitney U tests were used to determine group differences. Categorical variables were expressed as percentages (%), and Chi-square tests were used to compare differences.

To identify the factors that were substantially linked with either the BRI or the eGFR, Spearman correlation tests were run. We utilized logistic regression analysis to obtain the odds ratios (ORs) and 95% confidence intervals (CI) to assess the association between BRI quartile and low eGFR. We split BRI according to quartile cutoff points. The reference group was the bottom quartile. Model 1 was unadjusted. Model 2 adjusted for center, sex, and age. On the basis of Model 2, Model 3 further adjusted for drinking, smoking, diabetes, a history of coronary heart disease (CHD) and diabetes mellitus disease (DM). On the basis of Model 3, Model 4 additionally adjusted for FBG, TG, LDL-C, TC, SBP, and DBP. Make OR (95%CI) calculations for each model.

The study was carried out for gender, age (< 60/≥ 60 years), diabetes history, blood pressure, and smoking habits were stratified and adjusted for many potential confounders in order to examine the relationship between the BRI quartile and low eGFR in more detail. The ROC curve was then displayed, and the 95% CI and area under the receiver operating characteristic curve (AUCs) were reported to more clearly illustrate the predictive value of BRI and several anthropometric factors for low eGFR. SPSS Version 25.0 (IBM, Chicago, IL, USA) was used for data analysis, and MedCalc Version 13.0 was used for ROC analysis (MedCalc Software, Mariakerke, Belgium). In order for the findings to be considered statistically significant, the bilateral P values had to be less than 0.05.

## 3 Results

### 3.1 Clinical characteristics of study participants

After matching based on propensity scores, a total of 36,784 participants (11,546 men and 25,238 women) were enrolled in the study. According to whether or not the participants’ eGFR was raised, **Table 1** displays the clinical and biochemical characteristics of the subjects, who were divided into two groups. The low eGFR group had lower HDL-C, was older, had a higher prevalence of DM and CHD, and had higher LDL-C, WC, BMI, HbA1c, FBG, PBG, SBP, and DBP values than the normal eGFR group.

**Table 1:** Characteristics of the study population by eGFR category.

Variables	Before propensity score matching			After propensity score matching		
	Normal eGFR	Low eGFR	*P*-value	Normal eGFR	Low eGFR	*P*-value
n	39681	4256		32571	4213	
Age,years	57.00 ± 9.06	60.74 ± 10.08	<0.001	57.92 ± 9.00	60.11 ± 10.01	<0.001
Men,%	12301(31.0%)	1128(26.5%)	<0.001	10455(32.1%)	1091(25.9%)	<0.001
BMI,kg/m^2^	24.14 ± 3.66	24.67 ± 3.83	<0.001	24.25 ± 3.62	24.63 ± 3.80	<0.001
WC,cm	85.00(78.00,92.00)	87.00(80.00,94.00)	<0.001	85.00(78.00,92.00)	87.00(80.00,94.00)	<0.001
SBP,mmHg	130.00(117.00,144.00)	140.00(124.00,156.00)	<0.001	131.00(118.00,145.00)	140.00(124.00,156.00)	<0.001
DBP,mmHg	77.00(70.00,85.00)	79.00(72.00,88.00)	<0.001	77.00(70.00,85.00)	79.00(72.00,88.00)	<0.001
TC,mmol/L	4.95(4.21,5.71)	5.02(4.29,5.77)	0.006	4.98(4.25,5.73)	5.04(4.31,5.79)	0.004
TG,mmol/L	1.32(0.94,1.90)	1.54(1.08,2.22)	<0.001	1.36(0.98,1.93)	1.56(1.11,2.24)	<0.001
HDL,mmol/L	1.30(1.09,1.53)	1.23(1.04,1.46)	<0.001	1.31(1.10,1.55)	1.25(1.06,1.48)	<0.001
LDL,mmol/L	2.82(2.23,3.43)	2.92(2.34,3.54)	<0.001	2.83(2.24,3.45)	2.94(2.36,3.56)	<0.001
FBG,mmol/L	5.50(5.10,6.10)	5.77(5.20,6.90)	<0.001	5.51(5.11,6.12)	5.77(5.19,6.90)	<0.001
PBG,mmol/L	7.31(6.00,9.48)	8.51(6.60,12.20)	<0.001	7.30(6.00,9.46)	8.51(6.59,12.20)	<0.001
HbA1c,%	5.90 ± 0.30	6.10 ± 0.50	<0.001	5.90 ± 0.30	6.10 ± 0.50	<0.001
AST,U/L	20.00(17.00,24.00)	21.00(17.00,26.00)	<0.001	20.00(17.00,24.00)	21.00(18.00,26.00)	<0.001
GGT,U/L	20.00(14.00,31.00)	22.00(15.00,35.00)	<0.001	20.00(14.00,31.00)	21.00(15.00,35.00)	<0.001
UACR(mg/g)	8.51(5.38,14.16)	47.97(36.35,79.18)	<0.001	8.56(5.40,14.21)	47.89(36.31,79.02)	<0.001
BRI	3.95(3.19,4.81)	4.32(3.42,5.27)	<0.001	3.97(3.20,4.83)	4.33(3.43,5.29)	<0.001
eGFR,mL/min/1.73m^2^	116.63(105.62,130.14)	81.60(73.60,86.25)	<0.001	115.78(104.32,128.98)	81.61(73.60,86.26)	<0.001
Smoking habits, %			0.007			0.003
no	34125(86.0%)	3579(84.1%)		28076(86.2%)	3551(84.3%)	
occasionally	913(2.3%)	85(2.0%)		717(2.2%)	89(2.1%)	
usually	4643(11.7%)	592(13.9%)		3778(11.6%)	573(13.6%)	
Current drinker(%)			<0.001			<0.001
no	29880(75.3%)	3170(74.5%)		24591(75.5%)	3143(74.6%)	
occasionally	7222(18.2%)	796(18.7%)		5830(17.9%)	775(18.4%)	
usually	2579(6.5%)	290(6.8%)		2150(6.6%)	295(7.0%)	
Previous DM (%)	4206(10.6%)	775(18.2%)	<0.001	3485(10.7%)	728(18.1%)	<0.001
Previous CHD(%)	2380(6.0%)	272(6.4%)	<0.001	1987(6.1%)	270(6.4%)	<0.001

Data expressed as mean ± SD for continuous variables or median (IQR) for skewed variables and percentage (%) for categorical variables.

BMI, body mass index; WC, waist circumstance; SBP, systolic blood pressure; DBP, diastolic blood pressure; TC, total cholesterol; TG, triglyceride; HDL, high-density lipoprotein cholesterol; LDL, low-density lipoprotein cholesterol; FBG, fasting blood glucose; PBG, 2-h postload blood glucose; HbA1c, glycosylated hemoglobin; AST, aspartate transferase; GGT, gamma-glutamyl transferase; UACR, urinary albumin-creatinine ratio; BRI, body roundness index; eGFR, estimated glomerular fifiltration rate; DM, diabetes mellitus; CHD, coronary heart disease.

### 3.2 Correlation between BRI quartile and low eGFR

Multiple logistic regression analysis was done to investigate the connection between the BRI quartile and low eGFR (**Table 2**). Patients with higher BRI levels had a higher likelihood of having low eGFR in Models 1-3 compared to participants in the BRI first quartile (P < 0.001). Even after further adjusting for FBG, TG, LDL-C, TC, SBP, and DBP, the correlation for Model 4 remained significant (OR [95%CI] Q2 vs Q1 1.052 [1.021-1.091], OR [95%CI] Q3 vs Q1 1.189 [1.062-1.284], OR [95%CI] Q4 vs Q1 1.283[1.181-1.394], P-value for trend < 0.001).

**Table 2:** Association between BRI quartiles and eGFR in the total population.

Variables	BRI Quartiles				
	Q1	Q2	Q3	Q4	P-value for trend
Model 1					
OR(95%CI)	1	1.135(1.043-1.235)	1.373(1.266-1.490)	1.985(1.837-2.146)	
P value		0.004**	<0.001***	<0.001***	<0.001
Model 2					
OR(95%CI)	1	1.127(1.036-1.226)	1.359(1.251-1.475)	1.889(1.740-2.052)	
P value		0.005**	<0.001***	<0.001***	<0.001
Model 3					
OR(95%CI)	1	1.096(1.007-1.193)	1.306(1.202-1.420)	1.773(1.631-1.928)	
P value		0.035*	<0.001***	<0.001***	<0.001
Model 4					
OR(95%CI)	1	1.052(1.021-1.091)	1.189(1.062-1.284)	1.283(1.181-1.394)	
P value		0.046*	0.037*	<0.001***	<0.001

*: P-value <0.05; **: P-value <0.01; ***: P-value <0.001.

Model 1: unadjusted; Model 2: adjusted for age, sex, centres; Model 3: further adjusted for smoking habits, drinking habits, CHD history, and DM history based on Model 2; Model 4: additionally adjusted for FBG, TG, LDL, TC, SBP, and DBP based on Model 3; OR, odds ratio; CI, confidential interval; BRI, body roundness index; eGFR, estimated glomerular filtration rate, CHD, coronary heart disease; DM, diabetes mellitus; FBG, fasting blood glucose; TG, triglycerides; TC, total cholesterol; LDL, low-density lipoprotein cholesterol; SBP, systolic blood pressure; DBP, diastolic blood pressure.

### 3.3 The relationship between BRI and low eGFR in stratified analysis

After thoroughly controlling for age, sex, center, drinking and smoking habits, history of CHD, history of DM, FBG, TC, TG, LDL-C, SBP, and DBP, stratified analysis was utilized to further confirm the stability of the connection between BRI and low eGFR in various populations (**Table 3**). BRI in the fourth quartile in women was significantly linked with low eGFR compared to the first quartile (OR [95%CI] Q4vs Q1 2.427 [1.616-3.645], P-value for trend = 0.032), when gender was taken into account (P-interaction = 0.001). Men’s BRI of the third and fourth quantiles was significantly connected with low eGFR when compared to the first quantile (OR [95%CI] Q3vsQ1 1.724[1.122-2.648], OR [95%CI] Q4vsQ1 2.699 [1.484-4.908], P-value for trend = 0.001). When the subjects had no prior history of DM, the third and fourth quantiles of BRI were significantly correlated with low eGFR compared to the first quantile (OR [95%CI] Q3vsQ1 1.108 [1.002-1.278], OR [95%CI] Q4vsQ1 1.227 [1.011-1.346], P-value for trend = 0.008), according to the DM history stratification (P-interaction = 0.056). Participants who had a history of DM showed the similar tendency (OR [95%CI] Q3 vs Q1 1.131 [1.107-1.156] and OR [95%CI] Q4 vs Q1 1.522 [1.138-2.465], P-value for trend < 0.001).

**Table 3:** Association between BRI quartiles and eGFR in different participants.

Variable	BRI Quartiles					
	Q1 OR(95%CI),P value	Q2 OR(95%CI),P value	Q3 OR(95%CI),P value	Q4 OR(95%CI),P value	P-value for trend	P for interaction
Gender						<0.001
Women	1.0	1.200(0.653-2.205)	1.724(1.122-2.648)*	2.699(1.484-4.908)**	0.001	
Men	1.0	0.911(0.557-1.493)	1.043(0.549-1.982)	2.427(1.616-3.645)**	0.032	
DM history						0.056
No	1.0	0.818(0.643-1.042)	1.108(1.002-1.278)*	1.227(1.011-1.346)*	0.008	
Yes	1.0	0.874(0.8121.304)	1.131(1.107-1.156)**	1.522(1.138-2.465)**	<0.001	
Age, years						0.143
<60	1.0	0.767(0.335-1.755)	1.468(1.021-2.135)*	2.147(1.097-4.205)*	0.006	
≥60	1.0	1.085(0.706-1.669)	1.404(1.033-2.120)*	2.563(1.742-3.771)**	<0.001	
Blood pressure, mmHg						0.024
SBP<120 and DBP<80	1.0	0.858(0.286-2.571)	1.496(1.016-3.173)*	2.854(1.033-4.687)*	<0.001	
120≤SBP<140 and/or 80≤DBP<90	1.0	1.067(0.733-1.554)	1.692(0.865-3.253)	2.983(1.160-4.718)*	0.026	
SBP≥140 or DBP≥90	1.0	1.188(0.778-1.813)	2.306(1.489-3.572)***	3.677(1.842-6.697)***	<0.001	
Smoking habits						0.068
No	1.0	0.877(0.798-0.964)	1.132(1.003-2.134)*	1.394(1.054-1.845)*	0.002	
Occasionally	1.0	0.654(0.360-1.188)	0.931(0.053-1.699)	0.969(0.553-1.169)	0.320	
Usually	1.0	1.187(0.916-1.539)	1.305(1.004-1.697)*	1.583(1.069-2.132)*	0.004	

*: P-value <0.05; **: P-value <0.01; ***: P-value <0.001.

adjusted for age, sex, centres, smoking habits, drinking habits, CHD history, DM history, FBG, TG, LDL, TC, SBP, and DBP.

OR, odds ratio; CI, confidential interval; BRI, body roundness index; eGFR, estimated glomerular filtration rate; CHD, coronary heart disease; DM, diabetes mellitus; FBG, fasting blood glucose; TG, triglycerides; TC, total cholesterol; LDL, low-density lipoprotein cholesterol; SBP, systolic blood pressure; DBP, diastolic blood pressure.

Younger participants in Q3 and Q4 (age < 60 years) were more likely to experience a low eGFR when stratified by age (P-interaction = 0.143), compared with the first quartile of BRI (OR [95%CI] Q3 vs Q1 1.468[1.021-2.135], OR [95%CI] Q4 vs Q1 2.147[1.097-4.205], P-value for trend = 0.006). However, BRI in the third and fourth quartiles was significantly linked with low eGFR in participants who were older (age ≥ 60 years) (OR [95%CI] Q3 vs Q1 1.404[1.033-2.120], OR [95%CI] Q4 vs Q1 2.563[1.742-3.771], P-value for trend < 0.001).

Blood pressure stratification (P-interaction = 0.024) revealed a significant relationship between BRI and low eGFR in the third and fourth quartiles of the normal blood pressure group (OR [95%CI] Q3 vs Q1 1.496[1.016-3.173], OR [95%CI] Q4 vs Q1 2.854[1.033-4.687], P-value for trend < 0.001). Only BRI in the fourth quartile in the prehypertensive group showed a statistically significant relationship with low eGFR (OR [95%CI] Q4 vs Q1 2.983[1.160-4.718], P-value for trend = 0.026). Low eGFR was significantly linked with BRI in the third and fourth quartiles in the hypertensive group (OR [95%CI] Q3 vs Q1 2.306[1.489-3.572], OR [95%CI] Q4 vs Q1 3.677[1.842-6.697], P-value for trend < 0.001).

Smoking habit stratification revealed a significant correlation between BRI in the third and fourth quartile of nonsmokers and low eGFR (OR [95%CI] Q3 vs Q1 1.132[1.003-2.134], P-interaction = 0.068). There was no statistically significant relationship between BRI and low eGFR among occasional smokers (OR [95%CI] Q4 vs Q1 1.394 [1.054-1.845], P-value for trend = 0.002). BRI was significantly linked with low eGFR in the third and fourth quartiles of frequent smokers (OR [95%CI] Q3 vs Q1 1.305[1.004-1.697], OR [95%CI] Q4 vs Q1 1.583[1.069-2.132], P-value for trend = 0.002).

### 3.4 Anthropometric indicators and area under ROC of low eGFR

**Figure 2:**
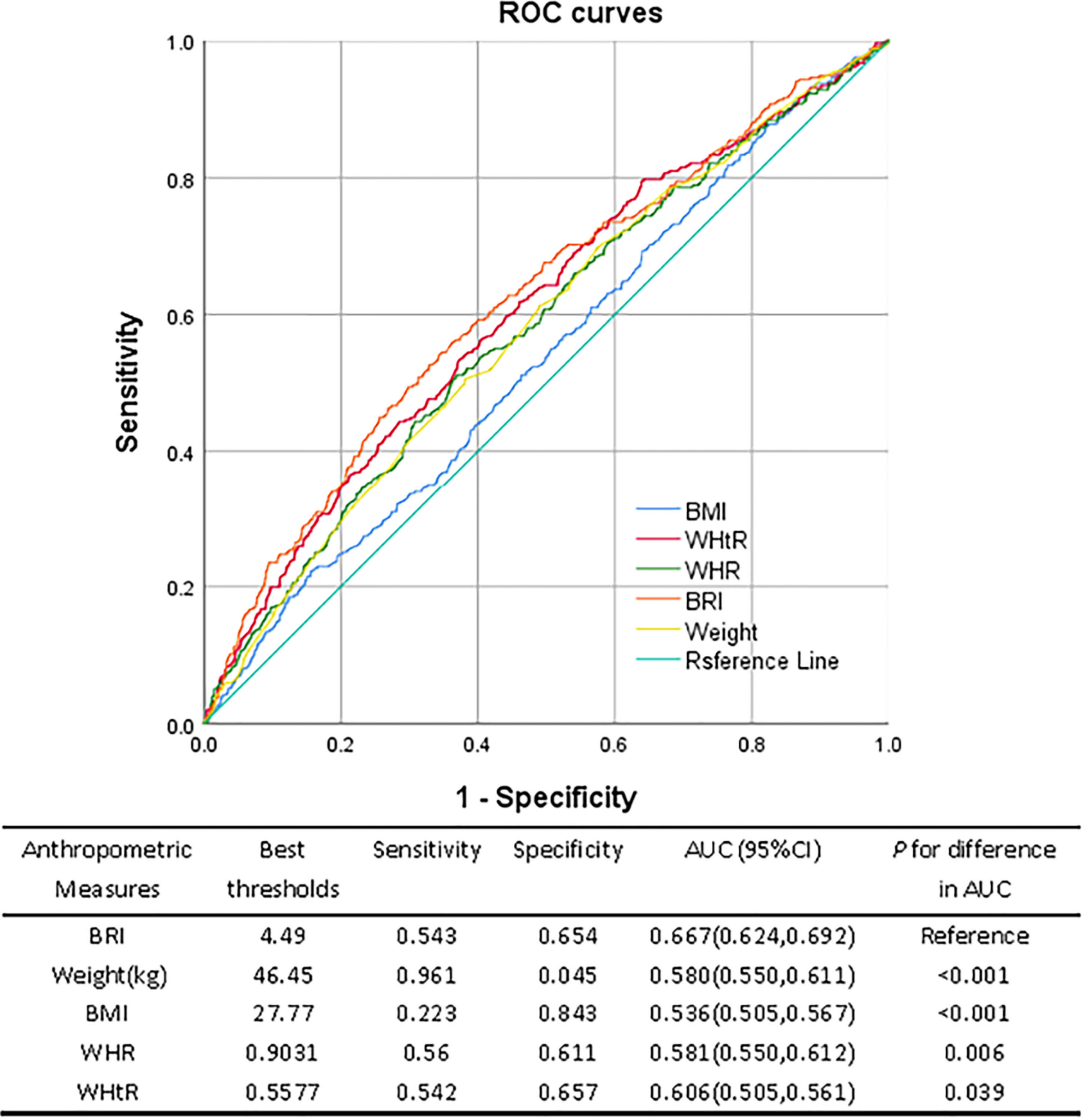
ROC curves of different anthropometric measures for discriminating low eGFR.

The ROC curves for several anthropometric indicators and the ideal cutoff values as determined by the Youden index are shown in **Figure 2**. The BRI’s predictive performance was significantly higher than BMI, WHtR, and WHR. The BRI’s cutoff value was 4.49, and its AUC was the largest (0.667, 95%CI: 0.624, 0.692), sensitivity was 54.3%, and specificity was 65.4%. The optimum cutoff for WHtR is 0.557, AUC was 0.606 (95%CI: 0.505, 0.561), the sensitivity and specificity were 50.5% and 56.1% respectively. The optimum cutoff for WHR is 0.9031, AUC was 0.581 (95%CI: 0.550, 0.612), 56.0% and 61.1%, respectively, were the sensitivity and specificity. The maximum BMI cutoff was 27.77, AUC was 0.536 (95%CI: 0.505, 0.567), Specificity was 84.3%, and sensitivity was 22.3%. When the body weight cutoff value was 46.45 kg, the AUC was 0.580 (95%CI: 0.550, 0.611), sensitivity was 96.1%, and specificity was 0.45%.

## 4 Discussion

In this investigation, we discovered a strong correlation between rising BRI levels and low eGFR. Importantly, the association was diminished after additional adjustments for drinking and smoking habits, FBG, TC, TG, SBP, DBP, history of CHD, and history of DM, indicating that drinking, smoking habits, abnormal glucose or lipid metabolism, and history of cardiovascular disease may increase the risk of low eGFR in patients. Further stratified analysis revealed that participants with higher BRI were more likely than participants with lower BRI to have low eGFR, particularly those who were older (≥ 60 years), women, and those with hypertension or DM history, as well as participants who regularly smoked. Additionally, we discovered that BRI had a stronger correlation with the low eGFR than other anthropometric variables as BMI, weight, WHtR, WHR, and had higher predictive power.

The prevalence of overweight and obese adults globally has increased dramatically over the past 30 years, which presents a serious challenge to public health due to the rapid economic development and changes in living patterns. The majority of overweight or obese people are now found in China, where one in five children and approximately half of adults are overweight or obese (11). Obesity can be categorized as systemic, central, or peripheral depending on where the fat deposits are located. However, there is growing interest due to the strong connection between central obesity and numerous disorders, including cardiovascular disease, DM, hypertension, CKD, and metabolic diseases.

The WHO recognizes BMI as an indicator of obesity, but it has limitations, including the inability to distinguish visceral fat from subcutaneous fat, similar to other conventional anthropometric indicators like WC, WHtR, and WHR (12). The new anthropometric index BRI was more accurate in predicting visceral adipose tissue than BMI (5) and WC (13). In DM patients, BRI is a useful marker of visceral fat accumulation (14). The metabolic syndrome (15), hyperuricemia (16), arteriosclerosis (13), and left ventricular hypertrophy (17) have all been demonstrated to be intimately related to BRI.

Obesity is linked to a number of harmful health effects, including a higher risk of CKD. The majority of earlier research, however, concentrated on the relationship between BMI and eGFR. BMI was found to be closely associated with eGFR decline among older adults (18). Martin et al. (19)discovered a similar significant positive correlation between elevated BMI and low eGFR in patients with CKD and obesity in Ireland. In a study of 75,000 participants, John Munkhaugen et al. discovered a significant link between BMI and the risk of kidney disease, with obese people being more likely to develop kidney disease (20).

Few studies have examined the relationship between visceral obesity and CKD, by exploring correlation between WHR or WC and GFR decline (21), or End stage renal disease (22), independent of BMI levels. High visceral adiposity index, a measure of visceral adiposity, has been linked to an increased risk of developing CKD in earlier studies (23). High WC (24) and lipid accumulation product (25) are linked to a quicker loss of GFR. The risk of low eGFR was independently linked with visceral adiposity as measured by BRI in our investigation, which comprised 36,784 Chinese people.

The relationship between BRI and low eGFR is currently unclear, the following theories are put forth: The metabolic demands placed on the kidneys may firstly rise as a result of obesity. Obese people experience compensatory hyperfiltration to satisfy the metabolic demands of weight gain (26). The kidneys are harmed by elevated glomerular pressure, it has been demonstrated that hyperfiltration is a reliable predictor can raise the danger of chronic CKD. Second, obesity’s complicated endocrine activity, which is characterized by the production of leptin (27), resistin (28), and adiponectin (29), can also cause direct kidney injury, causing oxidative stress (30), abnormal lipid metabolism (31), renin-angiotensin-aldosterone system activation (32), chronic inflammation (33), and insulin resistance (34). Ectopic lipid accumulation (35), increased fat deposition in the renal sinuses (36), glomerular hypertension, increased glomerular permeability, impairment to the glomerular filtration barrier associated with ultrafiltration (37), GFR decline are the outcomes of these pathophysiological alterations. Third, obesity is a complicated metabolic disorder that can have a variety of adverse effects on several organ systems, including the kidneys (38), DM, and hypertension, both of which are risk factors for CKD and may mediate the effects of obesity on the kidneys, are linked to the development or exacerbation of obesity. Sex hormones may have an impact on how fat is distributed. After menopause, lower estrogen levels in women are substantially linked to obesity and more visceral fat accumulation (39). Age is known to be an independent risk factor for renal impairment (40). Older adults over 60 years of age are more prone to low estimated glomerular filtration than middle-aged.

The study found that patients who were overweight at age 20 had a three-fold increased risk of subsequent new kidney disease, even after adjusting for high blood pressure and diabetes. Excess fat may also increase the risk of DM, high blood pressure and atherosclerosis, which can indirectly lead to CKD (41). Considering that the trend of obesity is getting younger and younger, we have to pay attention to its damage to the multi-organ function of the whole body as well as the challenge to national health and economic pressure. The good news is that CKD brought on by obesity is largely preventable. Weight-loss therapies consistently reduced blood pressure, glomerular ultrafiltration, and albuminuria in obese CKD patients, according to a meta-analysis (42). Obesity and renal disease can be significantly avoided with adequate nutrition, exercise, and education about the risks associated with being overweight (43). Our study demonstrates the utility of BRI as a trustworthy, user-friendly, and efficient screening method to determine the population’s elevated risk of low estimated glomerular filtration in subsequent clinical practice.

This study has the advantage of being the first multi-center, large-sample, cross-sectional clinical analysis to examine the relationship between BRI and low eGFR. To the best of our knowledge. This study does, however, have certain limitations. First of all, In order to pinpoint the precise cause of the low eGFR and BRI, additional prospective cohort studies are needed because this study was cross-sectional. Secondly, the applicability of our findings to other regions and populations may be constrained because the participants we recruited were all from China and around 40 years old. Thirdly, because direct measurement of GFR is cumbersome, we used eGFR instead, but it must be considered that it may be affected by metabolism. Finally, the gold standard for determining the distribution of visceral obesity is considered to be magnetic resonance imaging and computed tomography. however, because of the radiation exposure risk and the time and cost associated with using them, we did not use them in our epidemiological investigations. However, previous studies have shown a strong correlation between BRI and VAT.

## 5 Conclusion

In this study, we discovered a statistically significant positive connection between rising BRI levels and low eGFR in adults from the Chinese population. A greater BRI level was associated with a higher incidence of low estimated glomerular filtration in women, the elderly, habitual smokers, those with a history of diabetes or hypertension. In comparison to BMI, WHtR, and WC, it has also been demonstrated that BRI may be the best anthropometric marker for predicting low eGFR. BRI may therefore be a helpful clinical indicator of chronic kidney disease in Chinese adults since it is a marker of visceral fat. We propose BRI as a quick and effective screening method to identify persons at high risk of low estimated glomerular filtration and advise them to adjust their lifestyles and engage in regular exercise in light of the association between obesity, particularly visceral fat, and CKD.

## Data availability statement

The original contributions presented in the study are included in the article/supplementary material. Further inquiries can be directed to the corresponding author.

## Ethics statement

The studies involving human participants were reviewed and approved by The Committee on Human Research of the Shanghai Jiao Tong University School of Medicine’s associated Rui-Jin Hospital. The patients/participants provided their written informed consent to participate in this study. Written informed consent was obtained from the individual(s) for the publication of any potentially identifiable images or data included in this article.

## Author contributions

All authors have read and approved the final manuscript. YZ, WG, and RR analyzed the data and wrote the manuscript. YL and BL were of significant assistance in using and applying SPSS. AW, XT, LY, ZL, GQ, LC, QW, ZG, WW and GN provided advice and assistance, YM contributed by revising the article.
